# Supplementary material for: Overlap between Central and Peripheral Transcriptomes in Parkinson’s Disease but Not Alzheimer’s Disease
Source: Int J Mol Sci. 2022 May 6;23(9):5200. doi: 10.3390/ijms23095200 (PMC9104085; doi:10.3390/ijms23095200)
Supplement: Supplementary file 1 [file ijms-23-05200-s001.zip › Supplementary Figures.pdf]

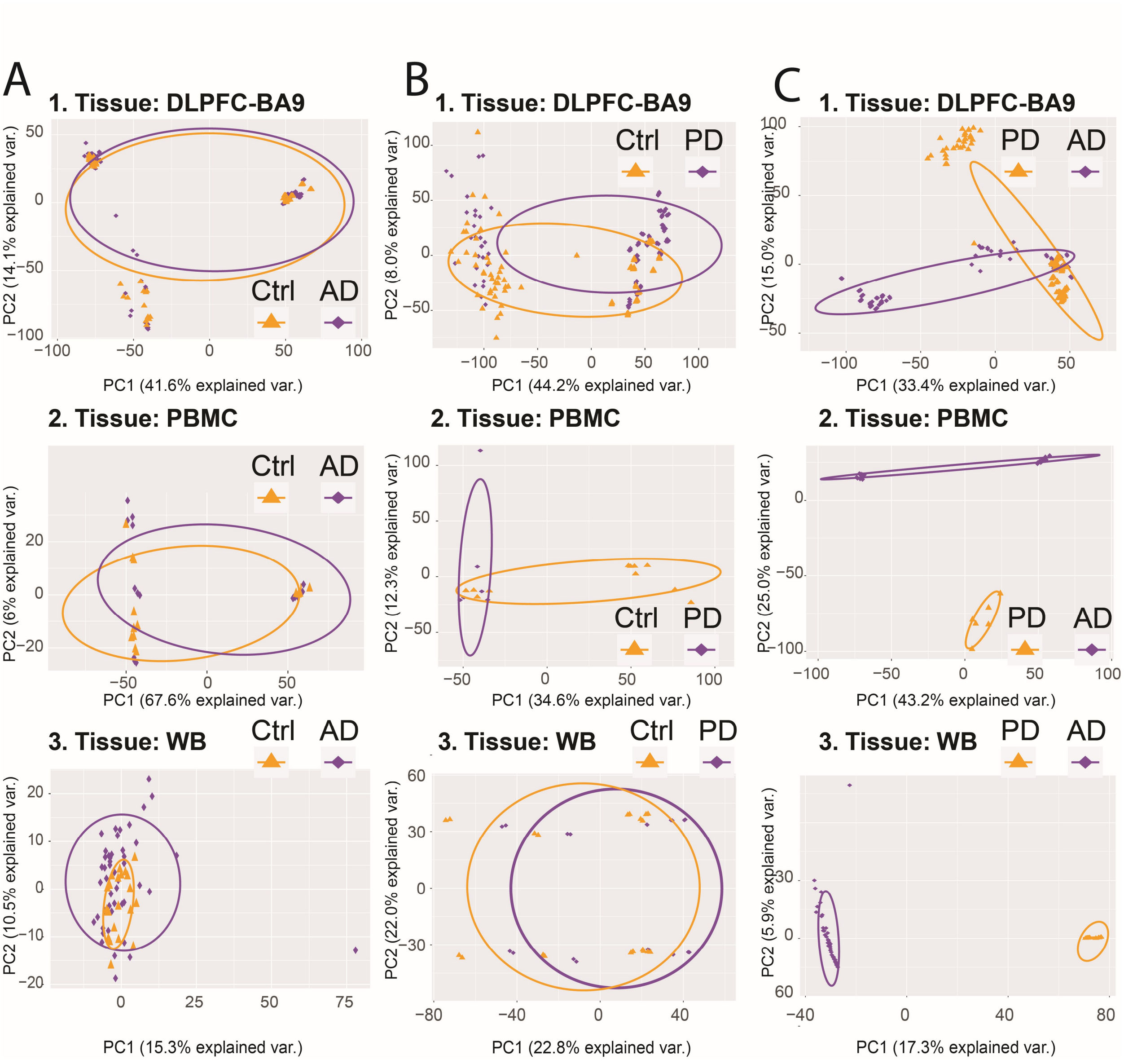

Figure S1: Score plots for the first two Principle Components (PCs). Graphs represent the separation of the sample populations of comparing groups **(A)** AD-vs-ctrl, **(B)** PD-vs-ctrl, and **(C)** AD-vs-PD) in different tissues **(1)** DLPFC-BA9, **(2)** PBMC, and **(3)** WB into clusters based on the first PC (PC1).

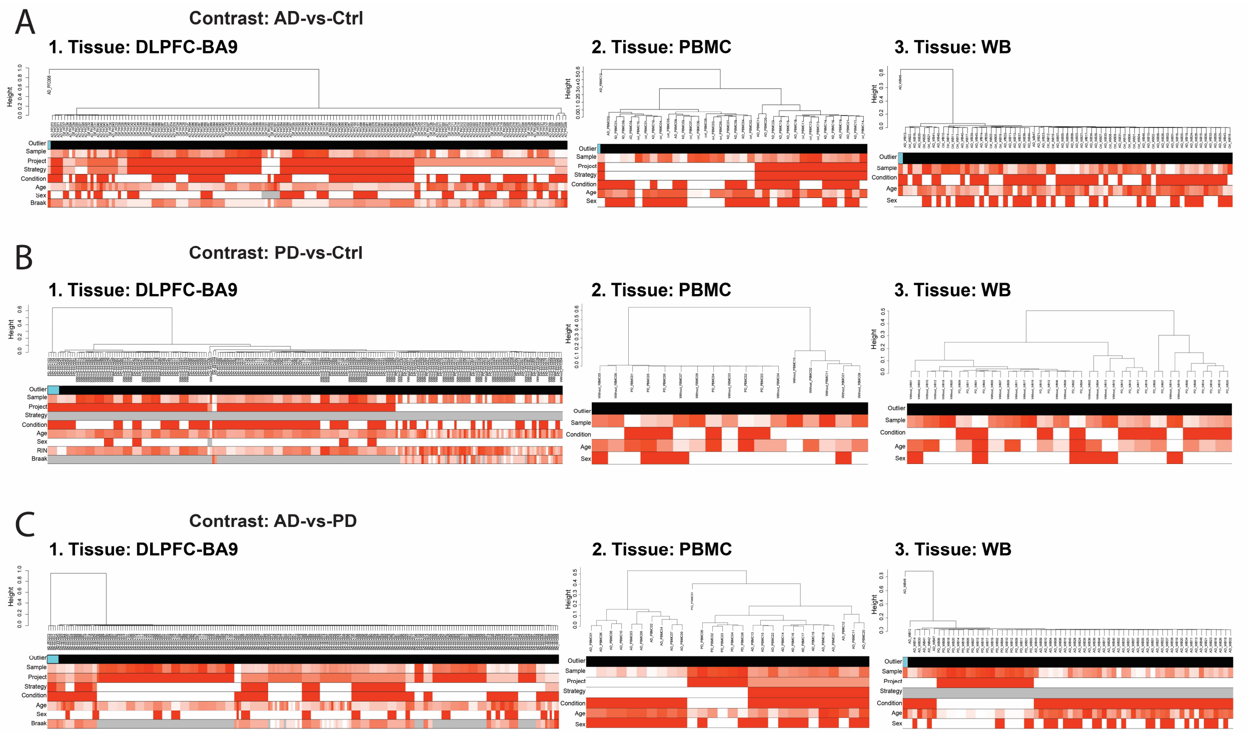

Figure S2: Cluster trees for **(A)** AD-vs-ctrl, **(B)** PD-vs-ctrl, and **(C)** AD-vs-PD) in different tissues **(1)** DLPFC-BA9, **(2)** PBMC, and **(3)** WB. The leaves of the tree correspond to the samples in each contrast. The first color band underneath the tree indicates which sample network appear to be outlying (Light blue box) compared to the ones that are grouped (solid black box). The remaining color-bands represent numerical values of physiologic traits (Red indicates high values).

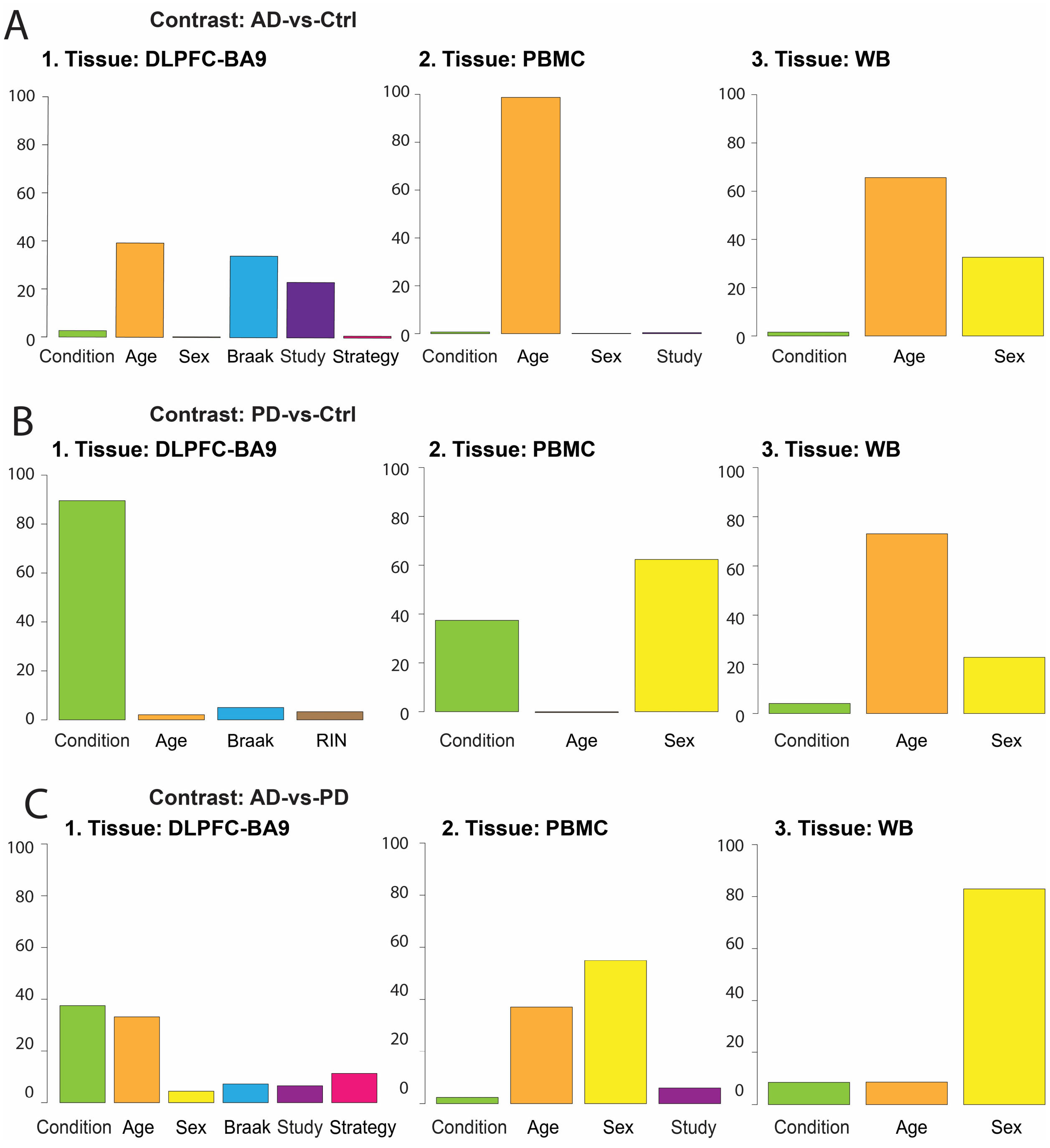

Figure S3: Schematic representation of ANOVA test results used to research the effects of multiple factors (Condition, Age, Sex, Braak, Study, Strategy) on expression data from the sample populations of comparing groups **(A)** AD-vs-ctrl, **(B)** PD-vs-ctrl, and **(C)** AD-vs-PD) in different tissues **(1)** DLPFC-BA9, **(2)** PBMC, and **(3)** WB. Y axis represents the percentage (%) of effectiveness.

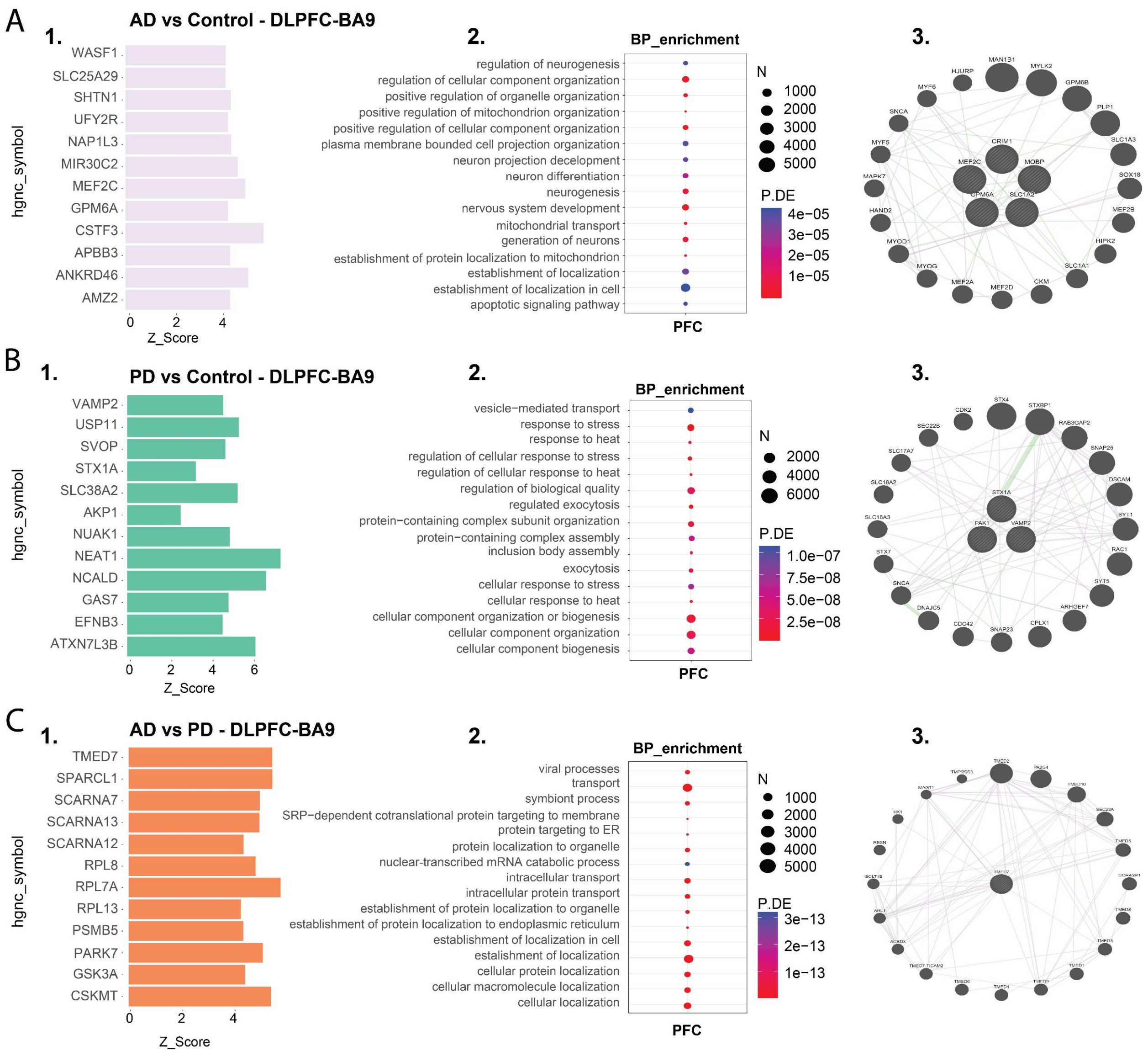

Figure S4: Schematic representation for DLPFC-BA9 tissue in comparing groups **(A)** AD-vs-ctrl; **(B)** PD-vs-ctrl, and **(C)** AD-vs-PD. **(1)** Top twelve features (gene) selected based on their MZS shown relative to the best diagnostic marker from the feature selection tool, Boruta. **(2)** GO-biological process (BP) associations according to their significance for AD, PD, and NC examined in the present study. In the plots, each dot's color and size represent adjusted p.value (P.DE) and number of genes (N), respectively. **(3)** Gene networks that interact (green) and are co-expressed (blue) with the selected gene biomarkers (genes in the middle) from the present study.

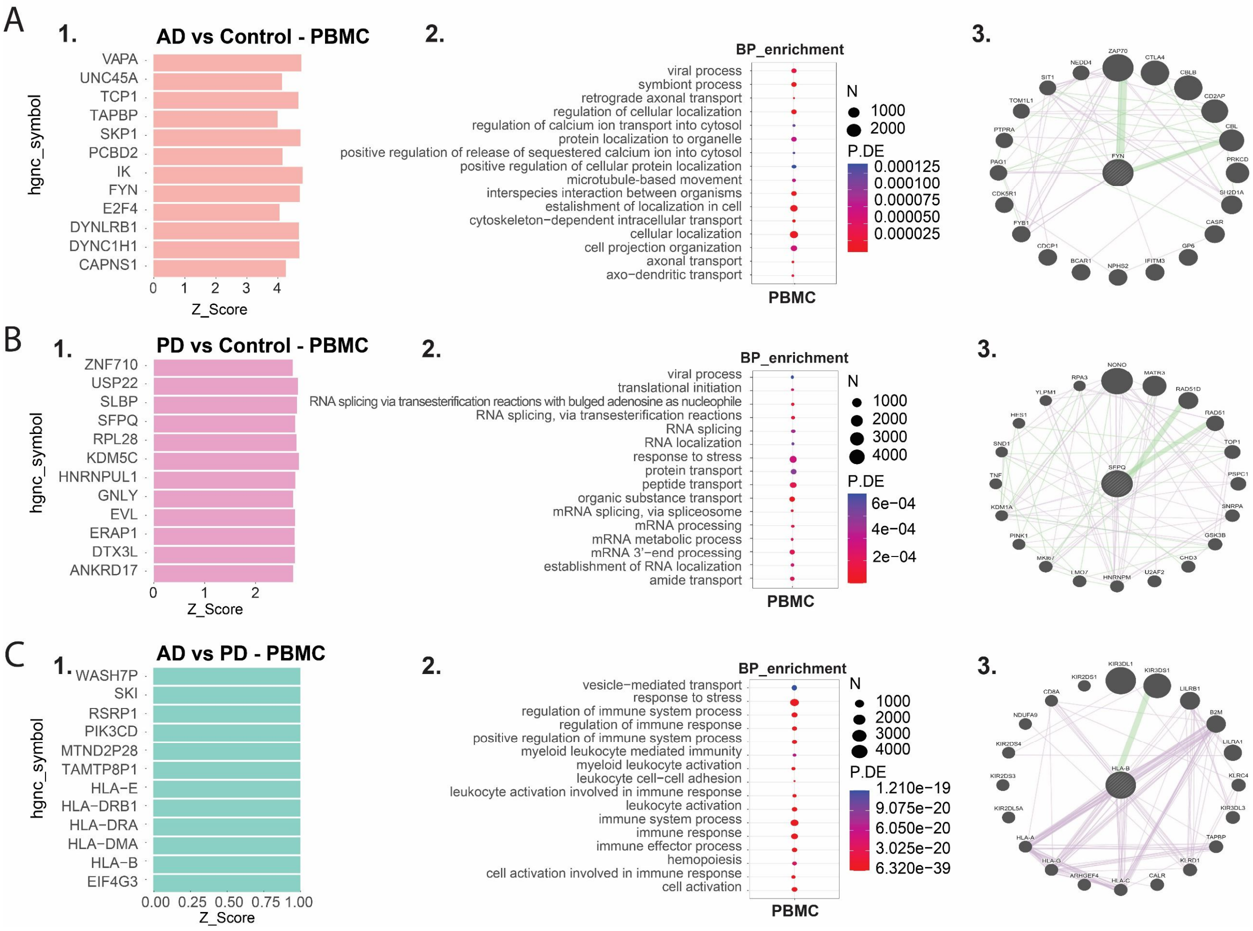

Figure S5: Schematic representation for PBMC tissue in comparing groups **(A)** AD-vs-ctrl **(B)** PD-vs-ctrl, and **(C)** ADvs-PD. **(1)** Top twelve features (gene) selected based on their MZS shown relative to the best diagnostic marker from the feature selection tool, Boruta. **(2)** GO-biological process (BP) associations according to their significance for AD, PD, and NC examined in the present study. In the plots, each dot's color and size represent adjusted p.value (P.DE) and number of genes (N), respectively. **(3)** Gene networks that interact (green) and are co-expressed (blue) with the selected gene biomarkers (genes in the middle) from the present study.

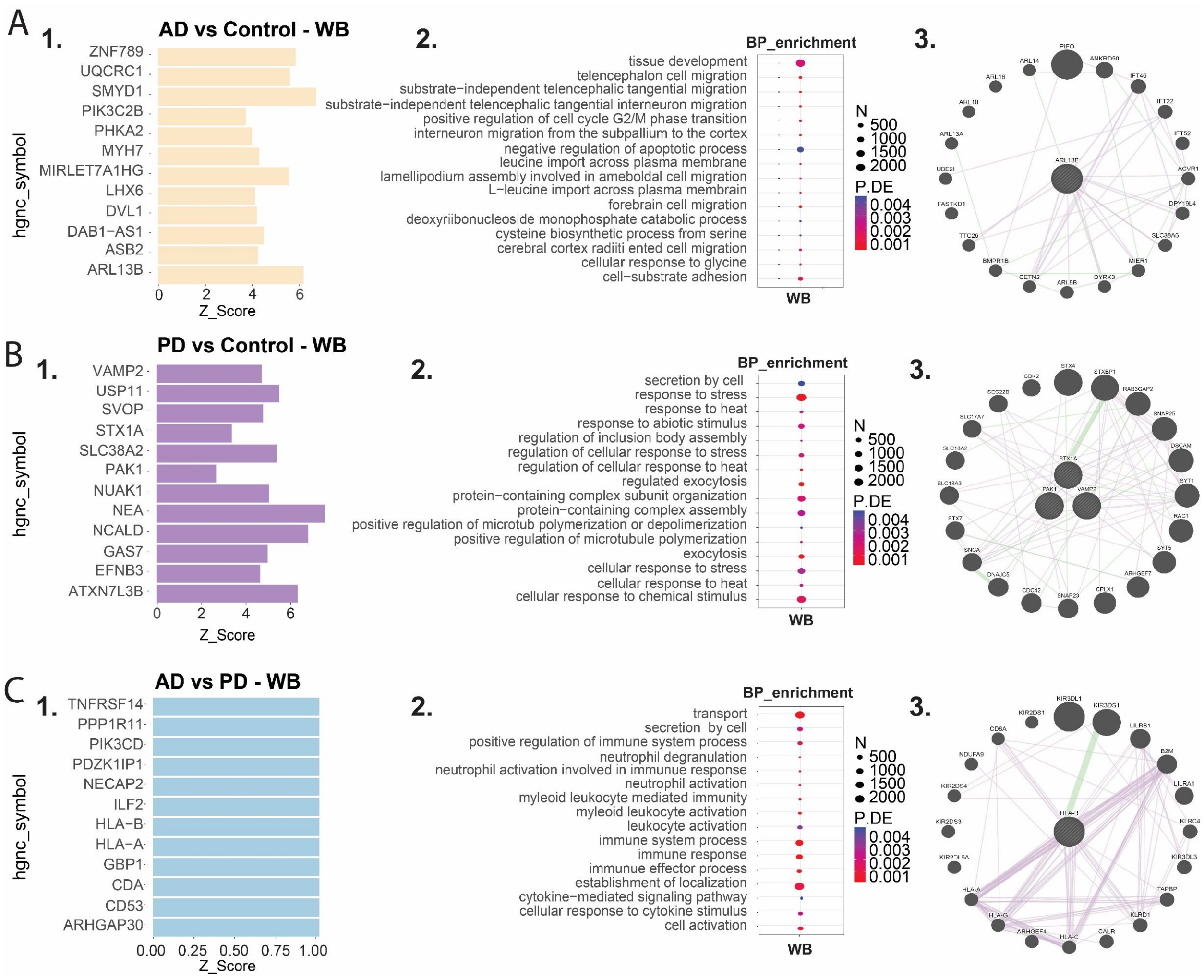

Figure S6: Schematic representation for WB tissue in comparing groups **(A)** AD-vs-ctrl **(B)** PD-vs-ctrl, and **(C)** AD-vsPD. **(1)** Top twelve features (gene) selected based on their MZS shown relative to the best diagnostic marker from the feature selection tool, Boruta. **(2)** GO-biological process (BP) associations according to their significance for AD, PD, and NC examined in the present study. In the plots, each dot's color and size represent adjusted p.value (P.DE) and number of genes (N), respectively. **(3)** Gene networks that interact (green) and are co-expressed (blue) with the selected gene biomarkers (genes in the middle) from the present study.
